# Biochanin A abrogates osteoclastogenesis in type 2 diabetic osteoporosis via regulating ROS/MAPK signaling pathway based on integrating molecular docking and experimental validation

**DOI:** 10.1186/s12906-023-04332-x

**Published:** 2024-01-08

**Authors:** Qi He, Junzheng Yang, Weijian Chen, Zhaofeng Pan, Baihao Chen, Jiaxu Zeng, Nenling Zhang, Yuewei Lin, Chuyi Chen, Jiacong Xiao, Miao Li, Shaocong Li, Haibin Wang, Peng Chen

**Affiliations:** 1 First School of Medicine, Guangzhou University of Chinese Medicine, 12 Jichang Road, Baiyun Area, Guangzhou, 510405 P.R. China; 2 The Laboratory of Orthopaedics and Traumatology of Lingnan Medical Research Center, Guangzhou University of Chinese Medicine, Guangzhou, 510405 P.R. China; 3 Fifth School of Medicine, Guangzhou University of Chinese Medicine, 12 Jichang Road, Baiyun Area, Guangzhou, 510405 P.R. China; 4 The State Key Laboratory of Functions and Applications of Medicinal Plants, School of Pharmaceutical Sciences, Guizhou Medical University, Guiyang, 550025 P.R. China; 5 Department of Orthopaedics, First Affiliated Hospital, Guangzhou University of Chinese Medicine, 12 Jichang Road, Baiyun Area, Guangzhou, 510405 P.R. China

**Keywords:** Biochanin A, Type 2 diabetic osteoporosis, Osteoclasts, Oxidative stress, MAPK signaling pathway

## Abstract

**Background:**

There are accumulating type 2 diabetes patients who have osteoporosis simultaneously. More effective therapeutic strategies should be discovered. Biochanin A (BCA) has been indicated that can play a role in improving metabolic disorders of type 2 diabetes and preventing osteoporosis. But whether BCA can treat type 2 diabetic osteoporosis has not been studied.

**Purpose:**

To investigate if the BCA can protect against type 2 diabetic osteoporosis and clarify the mechanism.

**Methods:**

Micro-CT and histology assays were performed to detect the trabecular bone and analyze the bone histomorphology effect of BCA. CCK-8 assay was performed to detect the toxicity of BCA. TRAcP staining, immunofluorescence and hydroxyapatite resorption assay were used to observe osteoclasts differentiation and resorptive activity. Molecular docking provided evidence about BCA regulating the MAPK axis via prediction by the algorithm. QRT-PCR and Western Blotting were utilized to detect the expression of osteoclastogenesis-related markers and MAPK signaling pathway.

**Results:**

Accumulation of bone volume after BCA treatment could be found based on the 3D reconstruction. Besides, there were fewer osteoclasts in *db/db* mice treated with BCA than *db/db* mice treated with saline. In vitro, we found that BCA hadn’t toxicity in osteoclasts precursor, but also inhibited differentiation of osteoclasts. Further, we found that BCA suppresses osteoclastogenesis via ROS/MAPK signaling pathway.

**Conclusion:**

BCA can prevent type 2 diabetic osteoporosis by restricting osteoclast differentiation via ROS/MAPK signaling pathway.

**Supplementary Information:**

The online version contains supplementary material available at 10.1186/s12906-023-04332-x.

## Introduction

Type 2 diabetic osteoporosis (T2DOP) is a kind of secondary osteoporosis (OP) induced by type 2 diabetes mellitus. It usually has no clinical symptoms in the early stage and is easy to be ignored, but it is prone to fracture in the later stage, which affects the quality of life of patients. Nearly 60% of patients with type 2 diabetes will develop OP. If patients are not treated in time, the mild cases may suffer from bone pain and functional limitation, and the severe cases may lead to disability, which brings a heavy economic burden to the family and society [1]. Therefore, the prevention and treatment of T2DOP have become an important problem to be solved urgently in the medical field.

Due to the complex pathological mechanism of T2DOP, there is no clear and effective treatment plan in clinical practice. At present, the treatment of T2DOP mainly relies on the combined intervention of hypoglycemic and anti-osteoporosis drugs [2]. However, there are still many shortcomings and limitations in these treatment measures, such as the excessive amount of medication, large adverse reactions, uncertain efficacy, long-term use and failure to ensure patient compliance [3]. Thus, the exploration of effective drugs for the treatment of T2DOP will provide an opportunity for clinical treatment. Traditional Chinese medicines (TCMs) can remarkably ameliorate T2DM and OP [4–6]. Among them, Yam, Pueraria and Astragali Radix (AR, Huangqi in Chinese) have been used in the treatment of T2DM and OP for more than 1500 years [7–9].

Biochanin A (BCA), an isoflavone present, is the main active component in Astragali Radix, Trifolium pratense L. and many other legume plants [10]. Biochanin A has been reported to have antioxidant, anti-inflammatory, neuroprotective, antiparasitic, anti-cancer and hepatoprotective activity [11, 12]. Some research indicated that BCA can provide cardiac protection by inhibiting the MAPK signaling pathway and growth of cardiac fibroblasts [13]. Besides, it can ameliorate inflammation by modulating NF-κB to block the expression and activity of pro-inflammatory cytokines. Many studies have shown that BCA not only has the effect of lowering cholesterol and hypolipidemic, but also can exhibit anti-hyperglycemic effects through decreasing insulin resistance and improving lipid metabolism to increase liver glycogen levels [14]. The hypoglycemic effects were further reconfirmed in type 2 diabetic models [15]. In addition, various studies showed that BCA can effectively prevent osteoporosis caused by ovariectomy, possibly by influencing the activity of osteoblasts and osteoclasts [16]. Thus, it can be expected that this compound may become a potential new lead for T2DOP drug development in the near future.

Taken together, BCA could not only treat diabetes, but also have an anti-osteoporosis effect [17]. However, there are no systematic scientific reports of BCA in the treatment of T2DOP. Therefore, our study was planned to evaluate the effect of BCA in *db/db* mice that are a model of T2DOP and set out the molecular mechanism of underlying BCA based on integrating molecular docking and experimental validation that provide theoretical groundwork for extensive use of BCA in the treatment of T2DOP.

## Materials and methods

### Reagents

BCA (https://pubchem.ncbi.nlm.nih.gov/compound/5280373) of purity ≥ 98% was provided by Lemeitian Medicine co., Ltd (Chengdu, China). Alpha modified minimal essential medium (α-MEM), fetal bovine serum (FBS) and penicillin/streptomycin were obtained from Thermo Fisher Scientific (Scoresby, Australia). M-CSF and RANKL were purchased from PeproTech Inc. (USA). Santa Cruz Biotechnology (San Jose, USA) provided primary antibodies for p-ERK1/2 (sc-D1117), ERK1/2 (sc-E1717), NFATc1 (sc-G3014), Cathepsin K (sc-C0810), β-Actin (sc-47,778) and DAPI. Primary antibodies targeting p − JNK1/2 (9252 S), JNK1/2 (9252 L) and c-Fos (2250 S) were acquired from Cell Signaling Technology (Beverly, USA). Secondary antibodies were purchased from Bioss (Beijing, China). Tsingke Biological Technology (Beijing, China) supplied the oligo-dT primer. Accurate Biology (Guangzhou, China) provided the SYBR green master mix and the Evo M-MLV RT Kit. Cell counting kit 8 (CCK-8) was bought from Beyotime Biotechnology (Shanghai, China). TRAcP staining kit (Sydney, Australia) was purchased from Sigma Aldrich.

### Animal model

The group assignments were described as follow: wild-type group (C57BL/6J, *n* = 5, 9 weeks old), model group (*db/db* mice, *n* = 5, 9 weeks old) and BCA group (*db/db* mice, *n* = 5, 9 weeks old). All mice were offered by the Guangdong YaoKang Biotechnology Co., Ltd (SCXK (Yue) 2020-0054) and were maintained in Laboratory Animal Center, The First Affiliated Hospital of Guangzhou University of Chinese Medicine (SYXK (Yue) 2018-0092). After being adaptively fed with a chow diet and sterilized water for 1 week, mice of BCA group were treated and the other groups were treated with equal distilled water orally for 4 weeks. For collecting bone, all mice were sacrificed by intraperitoneal injection of 1% sodium pentobarbital (100 mg/kg) at 14 weeks old. All methods were carried out in accordance with ARRIVE guidelines and the whole animal experiment was approved by the Ethics Review Board of The First Affiliated Hospital of Guangzhou University of Chinese Medicine (Ethic NO. TCMF1-2021006). Animal care and procedures comply with the ethical guidelines issued by the International Scientific Committee on Experimental Animals (ICLAS).

### Micro-CT

The right femurs were harvested and fixed for 24 h in 4% paraformaldehyde (PFA). After 24 h, these samples were stored in PBS until ready for use. Micro-CT equipment (Skyscan1172, Bruker, Belgium) was exploited to scan the joints using the following parameters: 80 kV voltage, 100µA current, 0.4 degrees rotation step, and 0.5 mm aluminum filter and 5 μm slice thickness. 500 μm thickness of distal femur trabecular was identified as the volume of interest (VOI) for the bone histomorphometry quantitative analysis. The CT Analyzer software (Bruker micro-CT, Kontich, Belgium) was utilized to evaluate the following parameters: bone volume/tissue volume (BV/TV, %), trabecular number (Tb.N, 1/mm), trabecular spacing (Tb.Sp, mm), trabecular thickness (Tb.Th, mm) and structure model index (SMI).

### Bone histology assay

The left tibias had been extracted and kept for 24 h in 4% PFA. After 24 h, these samples decalcified with 0.5 M EDTA (pH 7.4) for 2 weeks. Then, the samples were embedded in paraffin and 6-µm thick longitudinal sections were prepared using microtomes. Then 6-µm thick sections were used for standard H&E and TRAcP staining, respectively. Finally, the sections were detected by an inverted microscope (Olympus America). Image J software was used to analyze bone histomorphologic parameters, including osteoclast surface/bone surface (Oc.S/BS).

### Isolation of bone marrow macrophages (BMMs) and cell culture

BMMs were extracted from the lower limb bone marrow of 4 weeks old C57BL/6J mice and *db/db* mice. Then, BMMs were cultured in complete α-MEM adding 10% FBS, 1% penicillin/streptomycin and 50 ng/ml M-CSF and incubated at 37 °C in a 5% CO_2_ atmosphere. The complete medium was replaced every two consecutive days and subculture were performed when cells were 80–90% confluent.

### CCK-8 assay

BMMs was determined to detect the effect of BCA on cell viability by using CCK-8 kit. BMMs were seeded in 96-well cell culture plates at 5 × 10^3^ cells/well density and then incubated in α-MEM with 10% FBS and 1% penicillin/streptomycin. After culturing for reaching 60% confluence, the cells were treated with different concentrations of BCA (0µM, 1.25µM, 2.5µM, 5µM, 10µM and 20µM) for 48 h. Cell viability was then determined by adding 10% CCK-8 solution and incubating at 37 °C for 2 h. The absorbance at 450 nm was measured on a Mutiskan GO plate reader (Thermo Fisher Scientific, Vantaa, Finland).

### TRAcP staining

To induce osteoclasts (OCs) differentiation, BMMs had been plated at a density of 5 × 10^3^ cells/well on a 96-well plate. After cells reaching 40% confluence, 50ng/ml RANKL and various concentrations of BCA (00µM, 1.25µM, 2.5µM, 5µM, 10µM) were added to induce the formation of OCs in complete α-MEM for 5 days. TRAcP staining was used to identify multi-nucleated osteoclasts. Then, cells were stained with TRAcP staining kit following the manufacturer’s description. The stained BMMs were captured microscopically (Leica DMI 3000 M, German).

### Detection of the cytoskeleton of osteoclasts

BMMs were seeded in confocal dishes at a density of 5 × 10^3^ cells/well with complete α-MEM and subsequently step performed after cells reaching 40% confluence. After being stimulated by 50ng/mL RANKL for 5 days in the presence or absence of different BCA concentrations (5µM and 10µM), and then cells were fixed in 4% paraformaldehyde (PFA) and permeabilized for 5 min in 0.1% of Triton X-100 for 30 min. Then, PBS containing 3% bovine serum albumin (BSA) was added in cells and stained for 45 min for using rhodamine-conjugated phalloidin with F-actin ring staining subsequently. Cell nuclei were identified by DAPI after being washed in PBS. Then, images were obtained using a Leica TCS SPEII confocal fluorescence microscope (Leica, Germany).

### Hydroxyapatite resorption assay

To measure the bone resorption activity of osteoclasts, 1 × 10^5^ BMMs cells were uniformly seeded into the 6-well collagen-coated plates (BD Biosciences). When cells reaching 60% confluence, complete α-MEM and 50ng/mL RANKL were used to stimulate the differentiation of BMMs for 3 days. And induced by 50ng/mL M-CSF and RANKL to form mature osteoclasts for 3 days. Afterward, cells are gently detached from the plates using a cell sterile scraper and plated into the 96-well hydroxyapatite-coated plate (Corning Osteoassay, Corning, NY) at a density of 5 × 10^3^ cells/well. 5 and 10 µM BCA were used to treat BMMs in complete α-MEM and 50ng/mL RANKL. 10% bleach solution was added for 10 min to erase cells and showed the resorbed hydroxyapatite regions. An inverted microscope (Leica, Germany) was performed for observation and the area of hydroxyapatite surface resorbed by osteoclasts was quantified using ImageJ software.

### Virtual molecular docking

Interactions between active biochanin A and core proteins were validated by virtual molecular docking. Biochanin A was downloaded in SDF format from PubChem (https://pubchem.ncbi.nlm.nih.gov). The compounds were imported into Chem3D software, and then the MM2 position in the calculation module was used to optimize the structure and minimize the energy of the compounds, and the output was in mol*^2^ format. The PDB format of the core genes domain was downloaded from the PDB database (http://www.rcsb.org/). The core proteins were initially processed with Pymol software for dehydrating, dephosphorylation and other operations. The core target proteins and small molecules were stored as files in the “PDBQT” format, and the proper docking sites were set, and the models with the best docking of each core target were further hydrotreated and charged with AutoDockTools 1.5.6 [18]. In order to define the binding site of the target protein, we utilized the “define and edit binding site” function in the Receptor-Ligand Interactions module of the DS2019 software. We specifically selected the option “From PDB Site Records” to accurately identify the binding site. Finally, the docking between the compound and the core target was eventually completed by running Autodock-Vina [19]. Simultaneously, Discovery Studio 2019 is used to find the docking site and calculate the LibDockScore [20, 21]. Next, the virtual screening results of core proteins were visualized by PyMOL software for molecular docking verification display. The binding energy of < 0 indicated the spontaneous binding of active ingredients to target proteins. The Vina binding energy of ≤ -5.0 kcal∙mol^−1^ and a LibDockScore of > 0 suggested a stable ligand-receptor binding [22]. The results of molecular docking of ligand-receptor complex were displayed in 3D and 2D interactions to evaluate the reliability of bioinformatics analysis and prediction. Moreover, we conducted an analysis of the ADMET properties of the compound Biochanin A using SWISSADME (http://www.swissadme.ch/) to evaluate its safety.

### Molecular dynamics (MD) simulation

Molecular dynamics (MD) simulations were used to verify the structural stability and conformational flexibility of protein-ligand complexes. MD simulations provide a powerful tool for predicting the motion of each atom of a protein, ligand, and the physics governing the interatomic interactions at a given time [23]. In order to further investigate the binding process between biochanin A and core proteins, respectively, molecular dynamics simulations of the combined ligand-receptor complexes were performed, and the Simulation and Standard Dynamics Cascade modules of Discovery Studio 2019 software were used to obtain the stand parameters, and the simulation process of the ligand. The molecular parameters of the ligand were obtained using the charmm36 position and the molecular parameters of the receptor protein were obtained using the charmm position. The receptor-ligand complex was solventized during the Solvation module calculations. Molecular dynamics simulations are then run, including 5 stages: Optimization (I Minimization), Optimization I (I Minimization2), Heating, Equilibration, and Simulation Sampling (Production). View the conformational change animation showing the changes during the kinetic simulation. Once the molecular dynamics calculations are complete, analyze the structural properties of the molecular dynamics trajectory through the Analyze Trajectory module, such as geometric properties (distances, angles, and dihedral angles), or the number of non-bonded interactions per simulation frame, RMSD and root mean square fluctuations (RMSF) between different conformations, and detection of non-bonded interactions formed between biochanin A and proteins [24, 25].

### Detection of intracellular ROS levels

Intracellular ROS levels were determined using the Reactive oxygen species assay kit according to the manufacturer’s instructions. BMMs were seeded in 12‑well plates at a density of 2 × 10^5^ cells/well and cells reaching 60% confluence. Then cells were treated with RANKL and different concentrations (5 and 10 µM) of BCA. Serum‑free medium was used to wash three times, then incubated with 10 µM dichloro‑dihydro‑fluorescein diacetate (DCFH‑DA) in the dark for 20 min. Cells were washed with serum‑free medium and observed at a magnification of ×100 under a fluorescence microscope at an excitation wavelength of 488 nm. To further quantify ROS levels in BMMs. After the same treatment, cells were analyzed via FACS LSRFortessaTM flow cytometer (BD Biosciences, Franklin Lakes, NJ).

### Quantitative Real-Time Polymerase Chain Reaction (qRT-PCR)

BMMs were seeded in 6‑well plates at a density of 3 × 10^5^ cells/well. After cells reaching 70% confluence, cells were treated for 48 h as described above. By using TRIzol Reagent, total RNA was extracted and measured quality by Nanodrop 2000 (Thermo Scientific, Rockford, IL, USA). 1000 ng of total RNA was used to synthesize cDNAs. For each target gene, the relative expression levels of mRNAs were calculated following the 2^−ΔΔCt^ method and using 18s as a housekeeping gene (primer sequences were listed in Table 1).

**Table 1:** Quantitative real-time PCR primer sequences

**Genes**	**Forward (5’-3’)**	**Reverse (5’-3’)**
***Acp5***	**GCGACCATTGTTAGCCACATACG**	**CGTTGATGTCGCACAGAGGGAT**
***Nfatc1***	**GGTGCCTTTTGCGAGCAGTATC**	**CGTATGGACCAGAATGTGACGG**
***V-ATPase-d2***	**GTGAGACCTTGGAAGTCCTGAA**	**GAGAAATGTGCTCAGGGGCT**
***Ctsk***	**CCAGTGGGAGCTATGGAAGA**	**AAGTGGTTCATGGCCAGTTC**
***Mmp9***	**CGTGTCTGGAGATTCGACTTGA**	**TTGGAAACTCACACGCCAGA**
***18s***	**TGGTTGCAAAGCTGAAACTTAAAG**	**AGTCAAATTAAGCCGCAGGC**

### Western blotting

BMMs were incubated at a density of 3 × 10^5^ cells/well in 6‑well plates and adhered for cells reaching 70% confluence. First, cells were treated for 48 h as described above. Cell proteins were extracted with 80 µl RIPA lysis buffer for 30 min on ice and quantified by using BCA assay. Then, the proteins were loaded in 10% SDS-PAGE and transferred to the PVDF filter membranes. The membranes were then incubated with 5% nonfat milk for 1.5 h at room temperature after by washing with Tris-Buffered Saline Tween 20 (TBST) buffer three times. The following primary antibodies: anti-NFATc1, anti-c-Fos, anti-V-ATPase-d2, anti-CTSK, anti-JNK 1/2, anti-p-JNK 1/2, anti-ERK 1/2, anti-p-ERK 1/2 and anti-β-actin (1:1000) were incubated with membranes overnight in a cold room. The next day, the membranes were rinsed with TBST 3 times for 5min each and underwent incubation at room temperature for 2 h with horseradish peroxidase–conjugated secondary antibodies (1:2000). Protein bands were visualized using a chemiluminescence HRP substrate and gel imaging system (Bio-Rad, Hercules, CA). The density of each band was quantified by image J software.

### Statistical analysis

Experiments were repeated at least three times. All statistical analyses and visualization were performed using GraphPad Prism (8.0.2). Data were performed by Independent-samples T test and expressed as means ± standard deviation (SD). Differences with *p*-value ≤ 0.05 were considered to be statistically significant.

## Results

### BCA attenuates bone loss of *db/db *mice

**Fig. 1:**
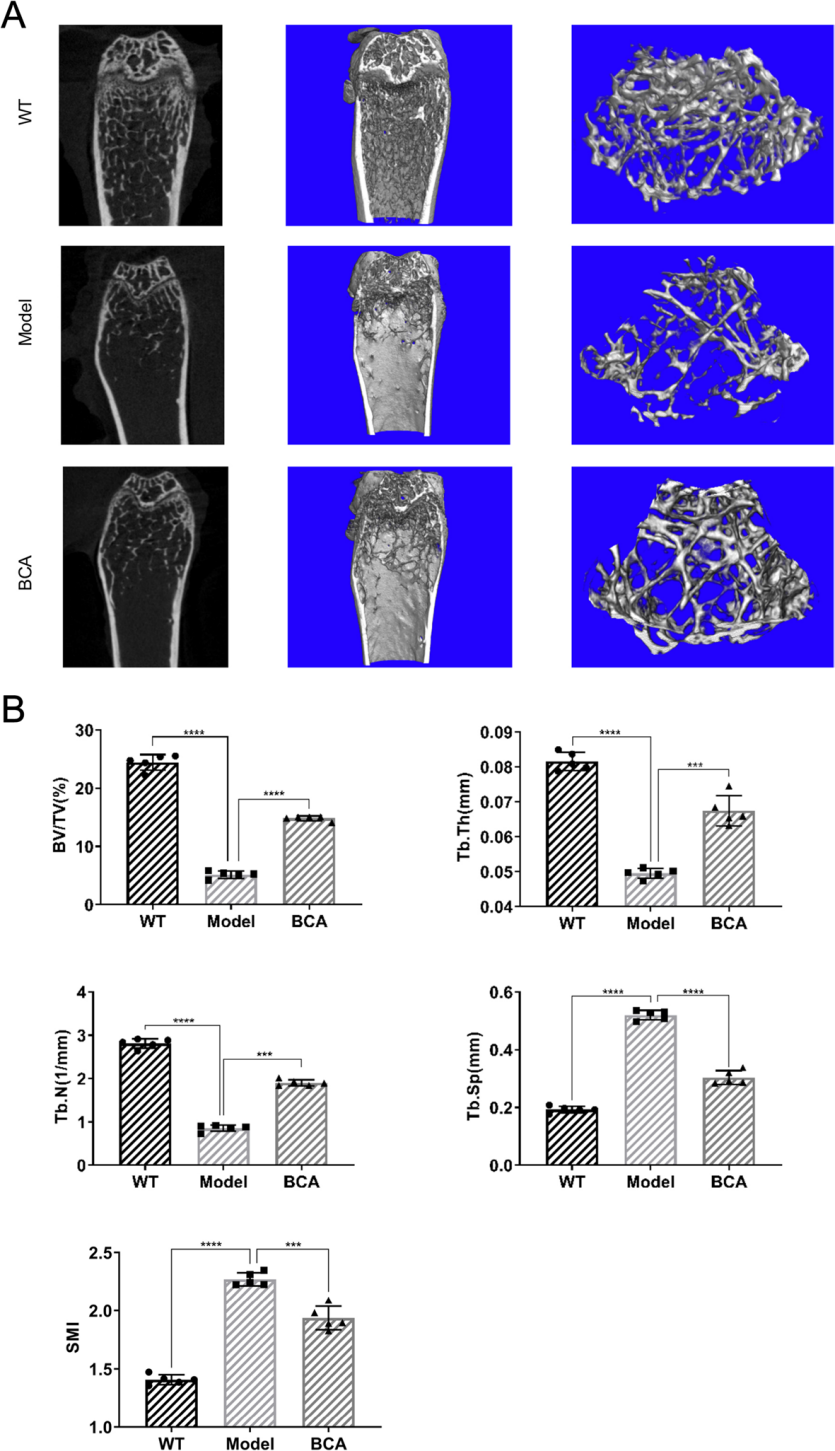
BCA improves bone loss in *db/db *mice. **A** Representative 2D and 3D reconstruction micro-CT figures of knee joints in WT, Model and BCA groups. **B **Quantitative measurements of tibia trabecular bone including BV/TV, Tb.N, Tb.Th, Tb.Sp and SMI. (Data were presented as mean ± SD. *n* = 5 per group. Significant differences between the Model and other groups were shown as * (*p*-value < 0.05), ** (*p*-value < 0.01), *** (*p*-value < 0.001) and **** (*p*-value < 0.0001))

**Fig. 2:**
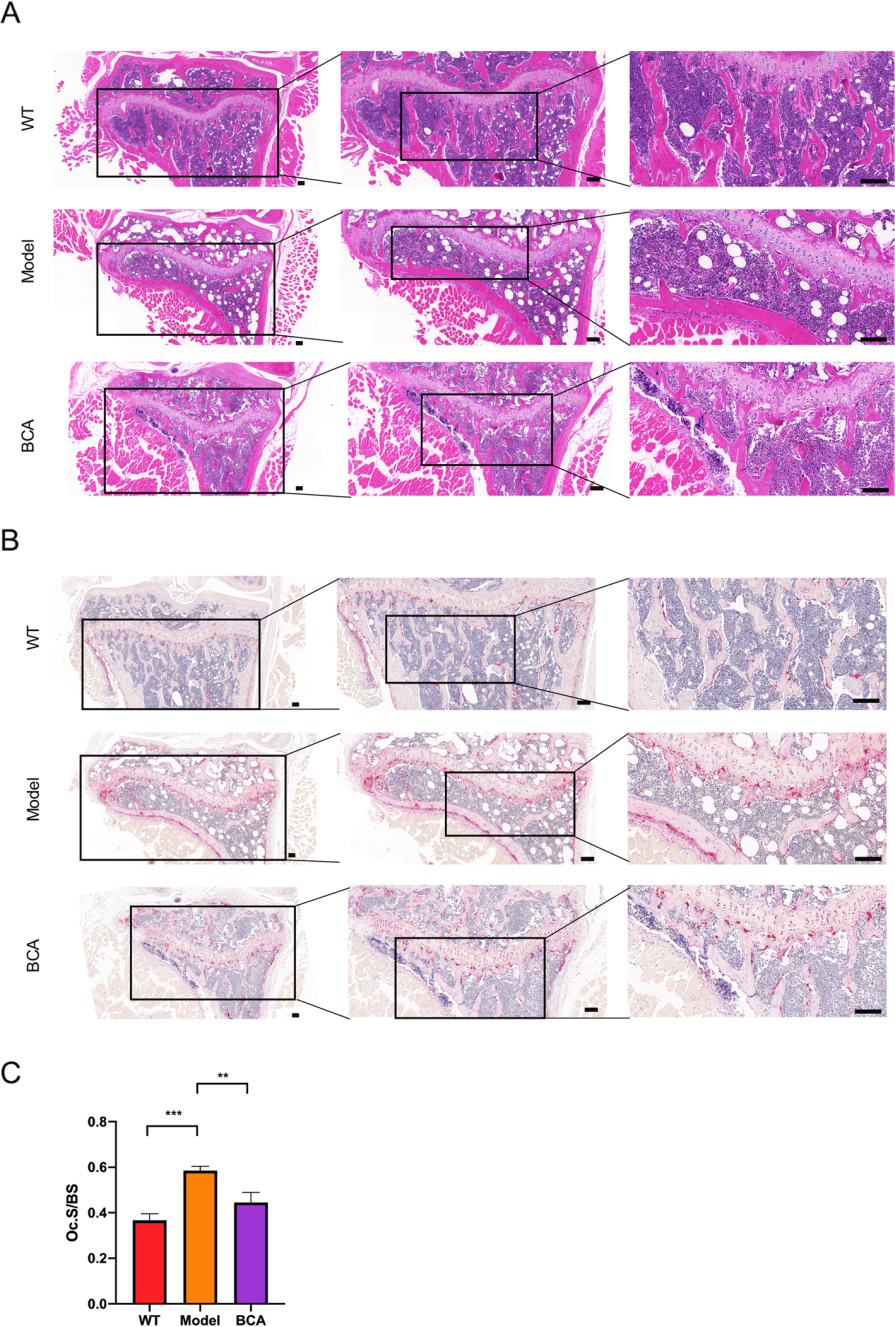
BCA attenuated histomorphological damage of murine *db/db*-induced osteoporosis with T2DM. Representative H&E (**A**) and TRAcP (**B**) staining images of the proximal tibia (Scale bars = 50 μm). **C** The quantifications of Oc.S/BS were calculated based on TRAcP staining by using the Image J software. (Data were presented as mean ± SD. Significant differences between the Model and other groups were shown as ** (*p*-value < 0.01), *** (*p*-value < 0.001))

To explore if BCA can improve type 2 diabetes-inducing bone loss, BCA was used to treat *db/db* mice. According to the results of micro-CT, we found that BCA can attenuate trabecular bone loss caused by type 2 diabetes (Fig. 1A). Besides, the quantitative analysis indicated that BCA significantly increases BV/TV, Tb.Th and Tb.N and decreases Tb.Sp and SMI (Fig. 1B). Compared with the WT group, H&E staining of the proximal tibia showed thinner, smaller trabeculae and more lipid droplets in the Model group (Fig. 2A). In contrast, we could find more normal trabecular bone in the BCA group compared to the Model group (Fig. 2A). Based on the results of TRAcP staining, much more TRAcP positive cells which are osteoclasts could be found in *db/db* mice bone (Fig. 2B, C). After treatment with BCA, osteoclasts were found to be decreased according to the values of Oc.S/BS (Fig. 2B, C). Altogether, these results significantly suggested that BCA was able to hold back histomorphological damage in *db/db* mice.

### BCA suppresses osteoclasts formation and activity

**Fig. 3:**
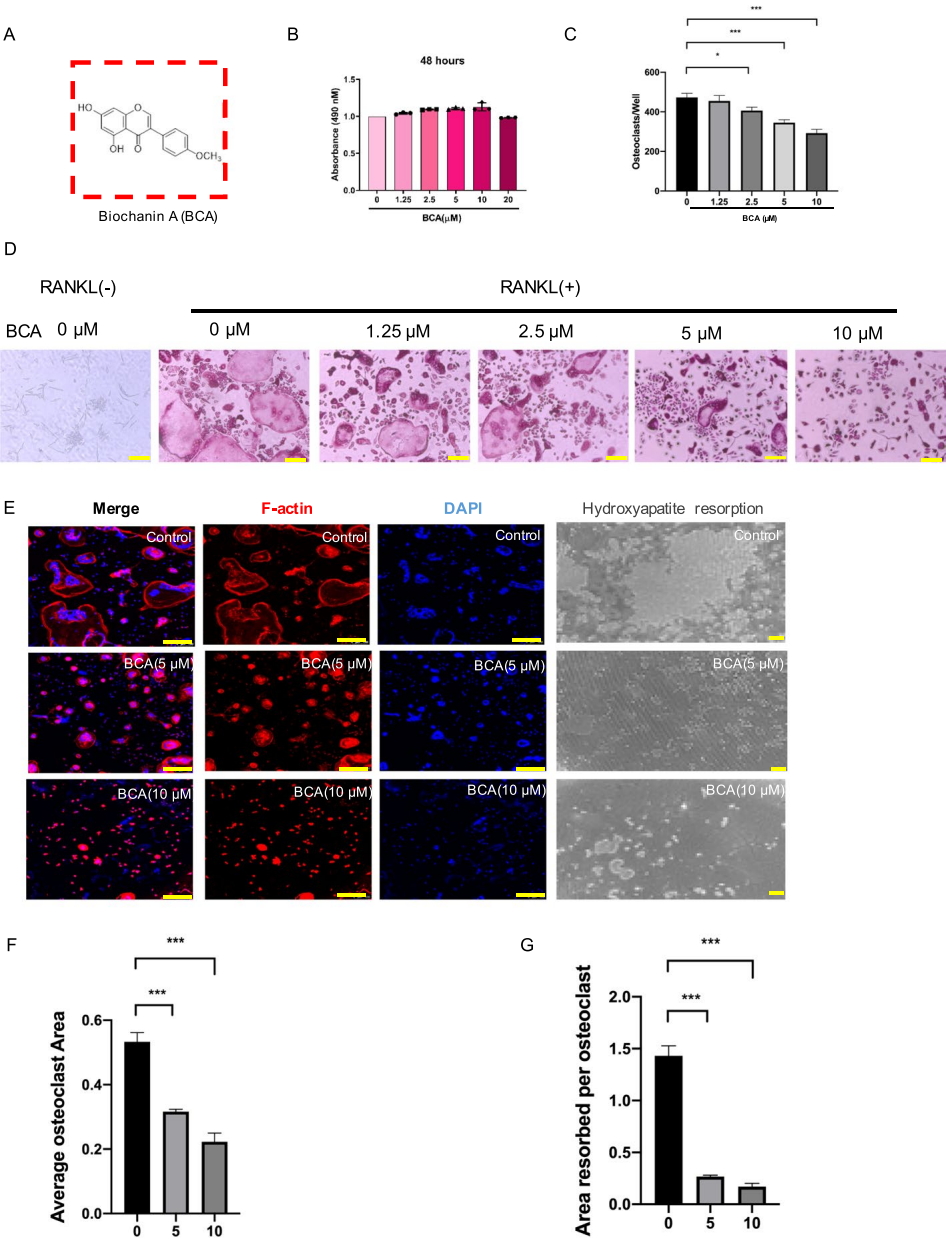
BCA abrogates RANKL-induced osteoclastogenesis in a dose-dependent manner. **A** Chemical structure of BCA. **B** The effects of the different concentrations of BCA (0, 1.25, 2.5, 5, 10 and 20µM) on BMMs for 48 h were measured by CCK-8 assay. **C **The number of osteoclasts per well (96-well plate) was quantitatively analyzed. TRAcP-positive multinucleated cells (> 3 nuclei) were identified as osteoclasts. **D **Representative images of TRAcP staining after treatment with BCA at increasing concentrations (Scale bars = 200 μm). **E **Representative confocal images of osteoclasts stained for F-actin belts (red) and nuclei (blue), and hydroxyapatite resorption images; the images include osteoclasts treated with RANKL and 0, 5 and 10µM BCA (Scale bars = 200 μm). **F **Quantitative analysis of osteoclasts in each well (96-well plate). **G **Quantitative analysis of resorbed proportion per osteoclast. (Data were presented as mean ± SD. Significant differences between the Model and other groups were shown as * (*p*-value < 0.05), *** (*p*-value < 0.001))

Based on the results of BCA treating animal model, we further detected whether BCA inhibited osteoclasts differentiation. Firstly, CCK-8 was performed to evaluate the toxicity of BCA (Fig. 3A, B). The safe concentrations including 1.25µM, 2.5µM, 5µM and 10µM were picked to measure the anti-osteoclastogenesis effect. The results showed that BCA suppresses osteoclasts formation in a dose-dependent manner (Fig. 3C, D). Thus, we identified 5µM and 10µM as the most optimal concentrations to finish the subsequent experiment. Osteoclast is characterized by multinuclear and obviously F-actin belt. Rhodamine-phalloidin and DAPI counterstaining were used to observe morphological changes of osteoclasts after treatment of BCA. We found that BCA can shrink F-actin belt of OCs and inhibit the aggregation of nuclei (Fig. 3E, F). In addition, RANKL exposed cells showed the active bone resorption status while breaking by various concentrations BCA (Fig. 3E, G).

### BCA decreases intracellular ROS levels of BMMs

**Fig. 4:**
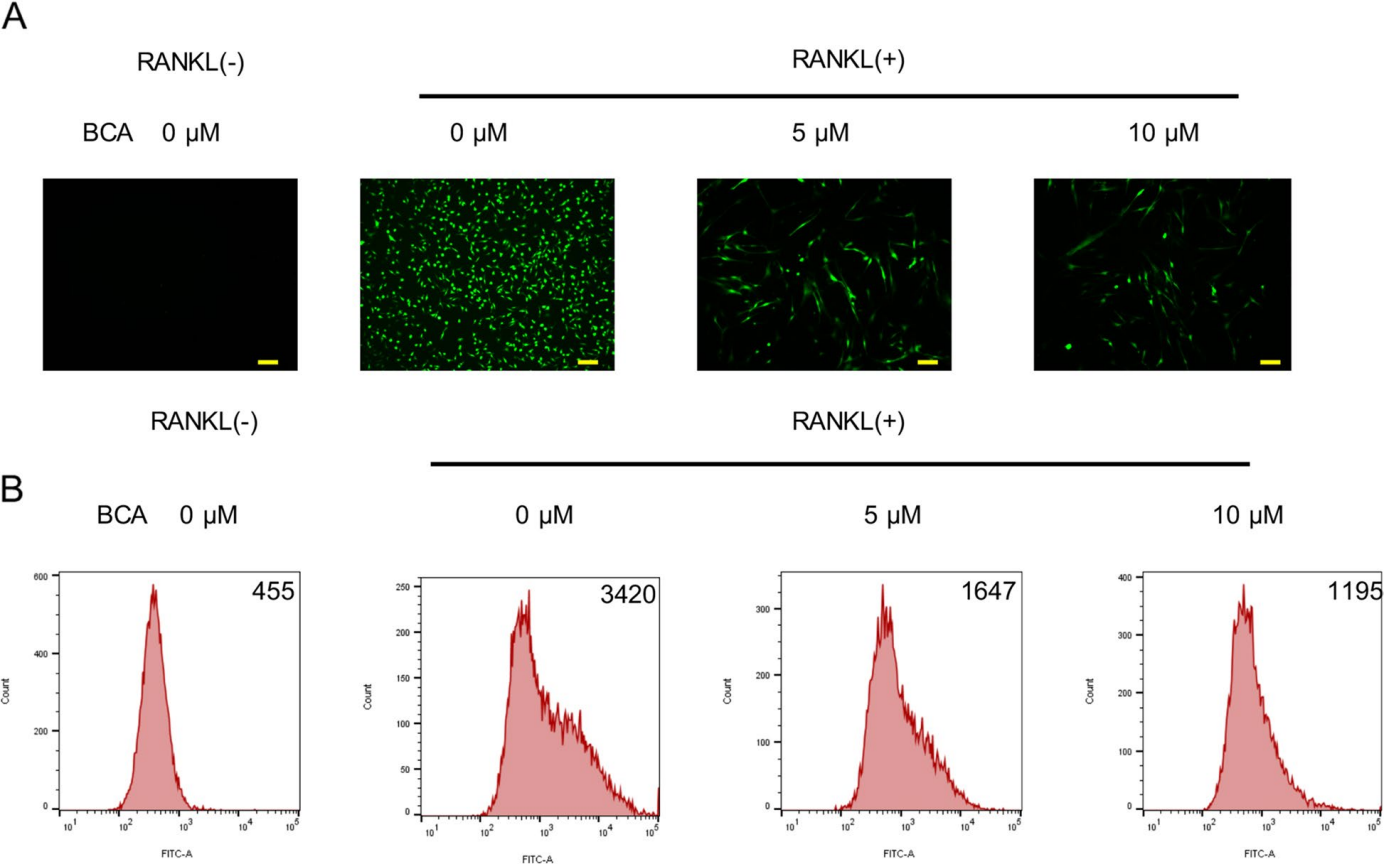
BCA reduces ROS levels induced by RANKL. **A **Representative fluorescence microscope images of BMMs stained for DCFH‑DA (Scale bars = 200 μm). **B** Representative histogram images of BMMs indicated that the mean fluorescence intensity was analyzed by flow cytometry; the images include osteoclasts treated with RANKL and 0, 5 and 10µM BCA

ROS accumulates with the process of OCs differentiation. We found that this process can be blocked by BCA. According to the results captured by fluorescence microscope and flow cytometer, the DCFH-DA fluorescence intensity significantly reduced after BCA treatment compared to the control group (Fig. 4A, B).

### BCA down-regulates osteoclastogenesis-related genes and proteins

**Fig. 5:**
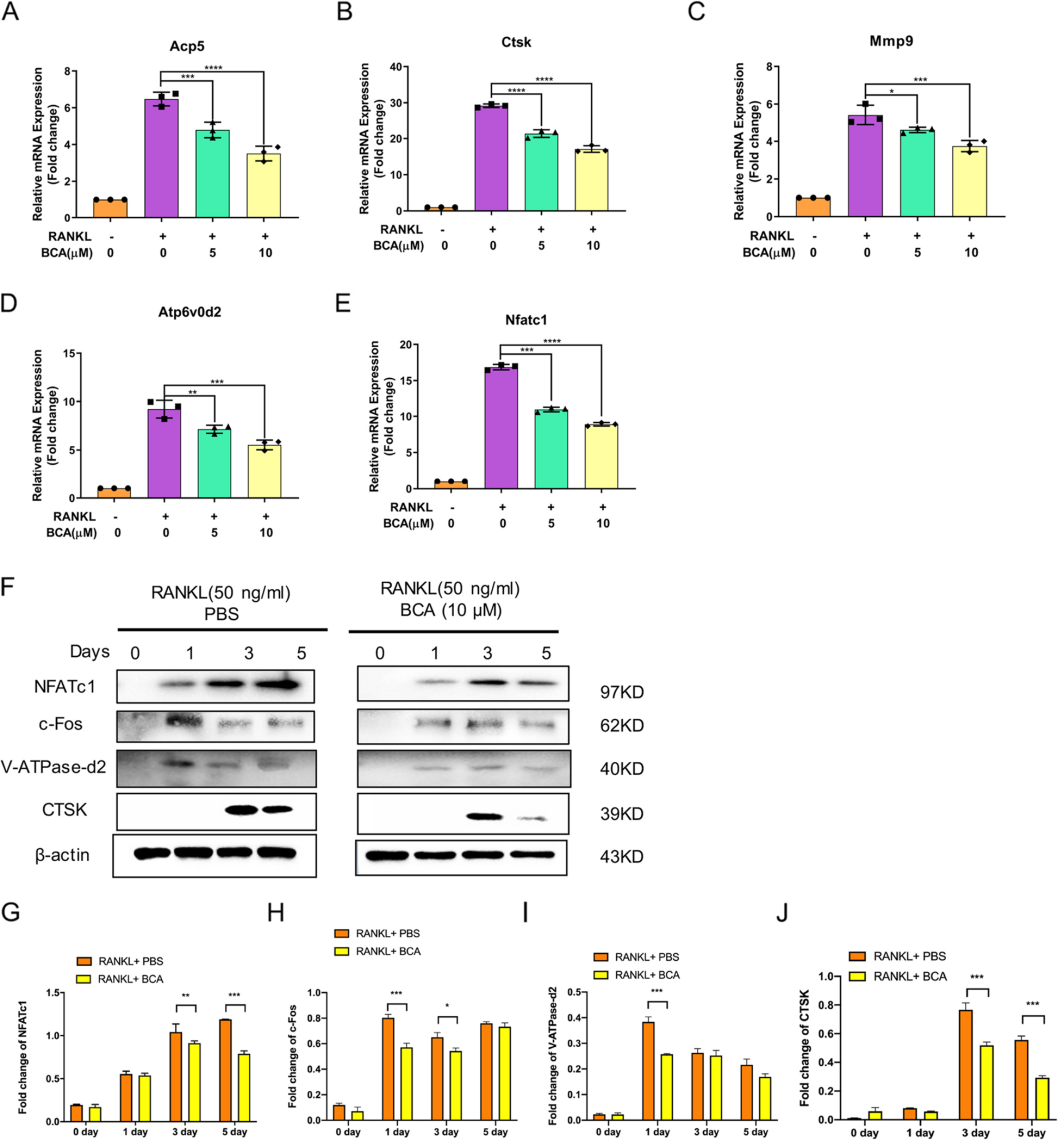
BCA blocks osteoclast-specific gene expression. **A **Acp5, (**B**) Ctsk, (**C**) Mmp9, (**D**) Atp6v0d2 and (**E**) Nfatc1 gene expression levels were standardized to 18s expression. (F) Representative western blotting images of the effects of BCA on NFATc1, c-Fos, V-ATPase-d2, and CTSK. **G**-**J **Quantitative analysis of the fold change after BCA (10µM) treatment. Data were presented as the means ± SD; *P < 0.05, **P < 0.01 and ***P < 0.001 relative to RANKL-induced controls

To further detect the effect that BCA regulating OCs differentiation, we evaluate the osteoclastogenesis-related mRNA expression including Acp5, Ctsk, Mmp9, Atp6v0d2 and Nfatc1. These RANKL-induced genes expression can be reduced by BCA in a dose-dependent manner (Fig. 5A). Furthermore, we demonstrated that BCA can down-regulate the expression of the osteoclastogenesis-related protein in a time-dependent manner. After treatment of 10µM BCA, RANKL-induced NFATc1, c-Fos, V-ATPase-d2, and CTSK were reduced across the process of OCs differentiation (Fig. 5F).

### Virtual molecular docking verification

**Fig. 6:**
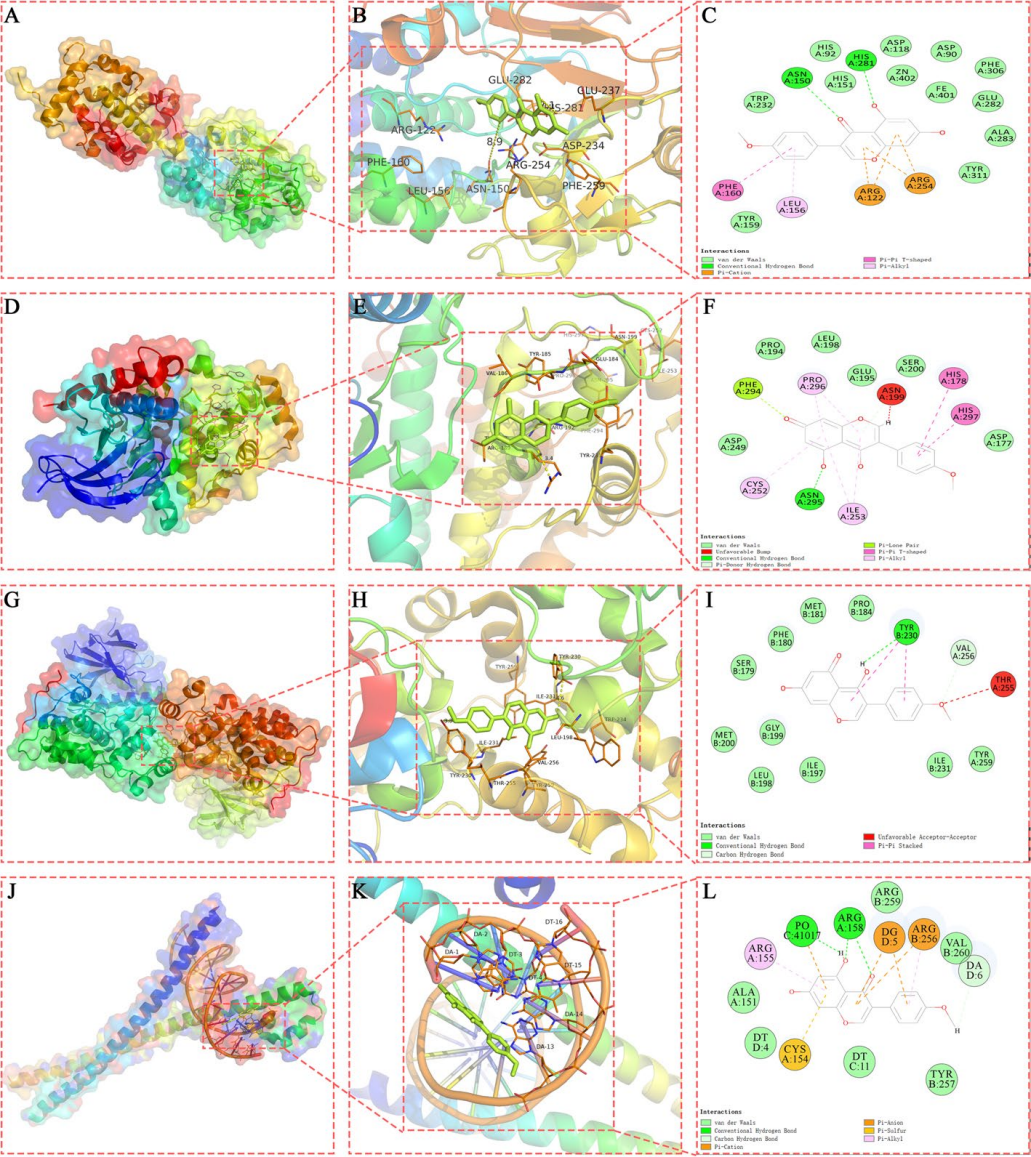
Molecular docking model of the biochanin A with the 3 core proteins. **A **NFATc1-BCA-3D (protein surface); (**B**) NFATc1-BCA-3D (cartoon model); (**C**) NFATc1-BCA-2D; (**D**) ERK-BCA-3D (protein surface); (**E**) ERK-BCA-3D (cartoon model); (**F**) ERK-BCA-2D; (**G**) JNK-BCA-3D (protein surface); (**H**) JNK-BCA-3D (cartoon model); (**I**) JNK-BCA-2D; (**J**) c-FOS-BCA-3D (protein surface) ; **K** c-FOS-BCA-3D (cartoon model); **L** c-FOS-BCA-2D

The active molecules BCA and the potential core proteins of osteoclast formation (NFATc1, ERK, JNK, NF-kB, P38, and c-FOS were selected for molecular docking verification. The results suggested that the binding energy of core protein P38 were greater than − 5.0 kcal·mol-1 in the docking model with the active component BCA. The binding energy of the docking model of BCA formed with core proteins NFATc1, ERK, JNK, and c-FOS was less than − 5.0 kcal·mol-1 and the value of RMSD was less than 2.00, which indicates that BCA can form more stable docking with proteins NFATc1, ERK, JNK and c-FOS than NF-kB, and P38. In addition, Discovery Studio 2019 software was used to dock with BCA and core proteins. As shown in Table 2, LibDockScore of docking models formed by NFATc1, ERK, JNK and c-FOS with BCA were all greater than 70. Among them, BCA formed hydrogen bond interaction with NFATc1 at HIS:281, ASN:150 amino acid sites in domain 5SVE, and formed hydrophobic interaction with NFATc1 at PHE:160, LEU:156, ARG:122, ARG:254 amino acid sites. Besides, BCA formed hydrogen bond interaction with protein ERK at MET:36, LYS:53, ALA:65, LYS:149, SER:151 amino acid site in domain 5UMO, and hydrophobic interaction was formed at LYS:52 amino acid site. The BCA and JNK formed a hydrogen bond interaction at TYR:230 amino acid site in structural domain 3O2M, and a hydrophobic bond at THR:255 amino acid site. BCA formed hydrogen bond interaction with protein c-fos at DA:6, PO:41,017, ARG:158 amino acid site in domain 2WT7, and hydrophobic interaction was formed at DG:5, CYS:154, ARG:155, ARG:256 amino acid site. Comprehensive analysis of the RMSD, the binding energy and the LibDockScore, the result showed that NFATc1 and BCA could form the most stable docking model. Every core protein and BCA form different hydrogen and hydrophobic bonds at different amino acid sites, they form different docking bodies. The results of the above molecular docking model were further verified by Pymol software (Fig. 6A-L). In addition, we performed an analysis of the properties of the compound BCA using SWISSADME. The results revealed that BCA exhibits moderate solubility, high gastrointestinal absorption, and high biological activity while demonstrating low toxicity. These characteristics make BCA a promising candidate for pharmaceutical applications (Supplementary Tables 1–6).

**Table 2:** Comparison of binding energy of molecular docking Autodock-Vina, and DS

**Protein**	**Compound**	**Structure**	**Vina(kcal·mol-1)**	**RMSD**	**DS(LibDockScore)**	**Hydrogen bond interaction**	**Hydrophobic interaction**
**NFATc1(5SVE)**	**biochanin A**		-6.2	1.801	93.5059	HIS:281,ASN:150	PHE:160,LEU:156,ARG:122,ARG:254
**ERK(5UMO)**	**biochanin A**		-5.2	1.459	79.1663	MET:36,LYS:53,ALA:65,LYS:149,SER:151	LYS:52
**JNK(3O2M)**	**biochanin A**		-8.1	1.059	87.5592	TYR:230	THR:255
**NF-kB(1RAM)**	**biochanin A**		-5.4	2.911	87.2753	ARG:84,ASP:153,ALA:156,ASP:80	LYS:79,PRO:82
**P38(6Y4T)**	**biochanin A**		-4.4	1.465	93.1559	MET:109,ALA:111	TYR:35,VAL:38,ALA:51,LYS:53,ALA:157
**c-fos(2WT7)**	**biochanin A**		-9.4	1.477	102.319	DA:6,PO:41,017,ARG:158	DG:5,CYS:154,ARG:155,ARG:256

### Molecular dynamics simulation

**Fig. 7:**
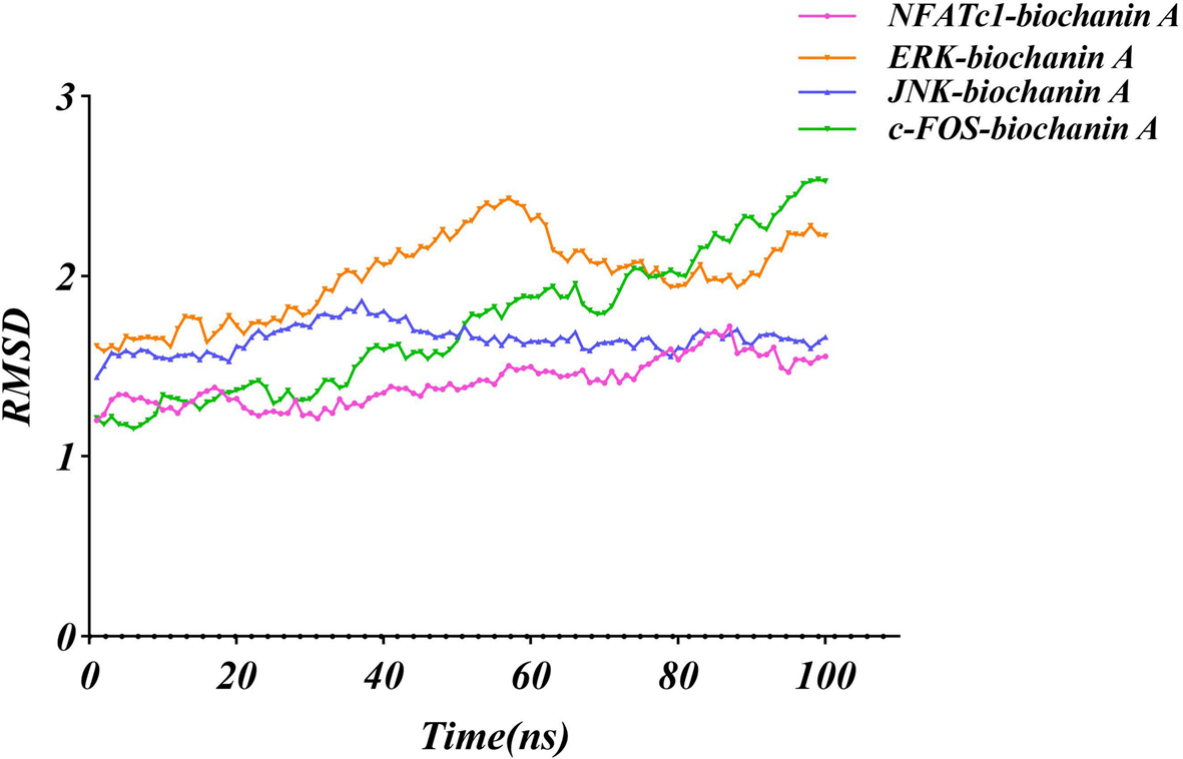
RMSD analysis of docked complex of NFATc1-BCA, ERK-BCA and JNK-BCA. (The purple line represents the change of RMSD value of NFATc1-BCA receptor-ligand complex; The orange line represents the change in RMSD of the ERK-biochanin A receptor-ligand complex; The blue line represents the RMSD changes of the JNK-BCA receptor-ligand complex; The green line represents the RMSD changes of the c-fos-BCA receptor-ligand complex)

**Fig. 8:**
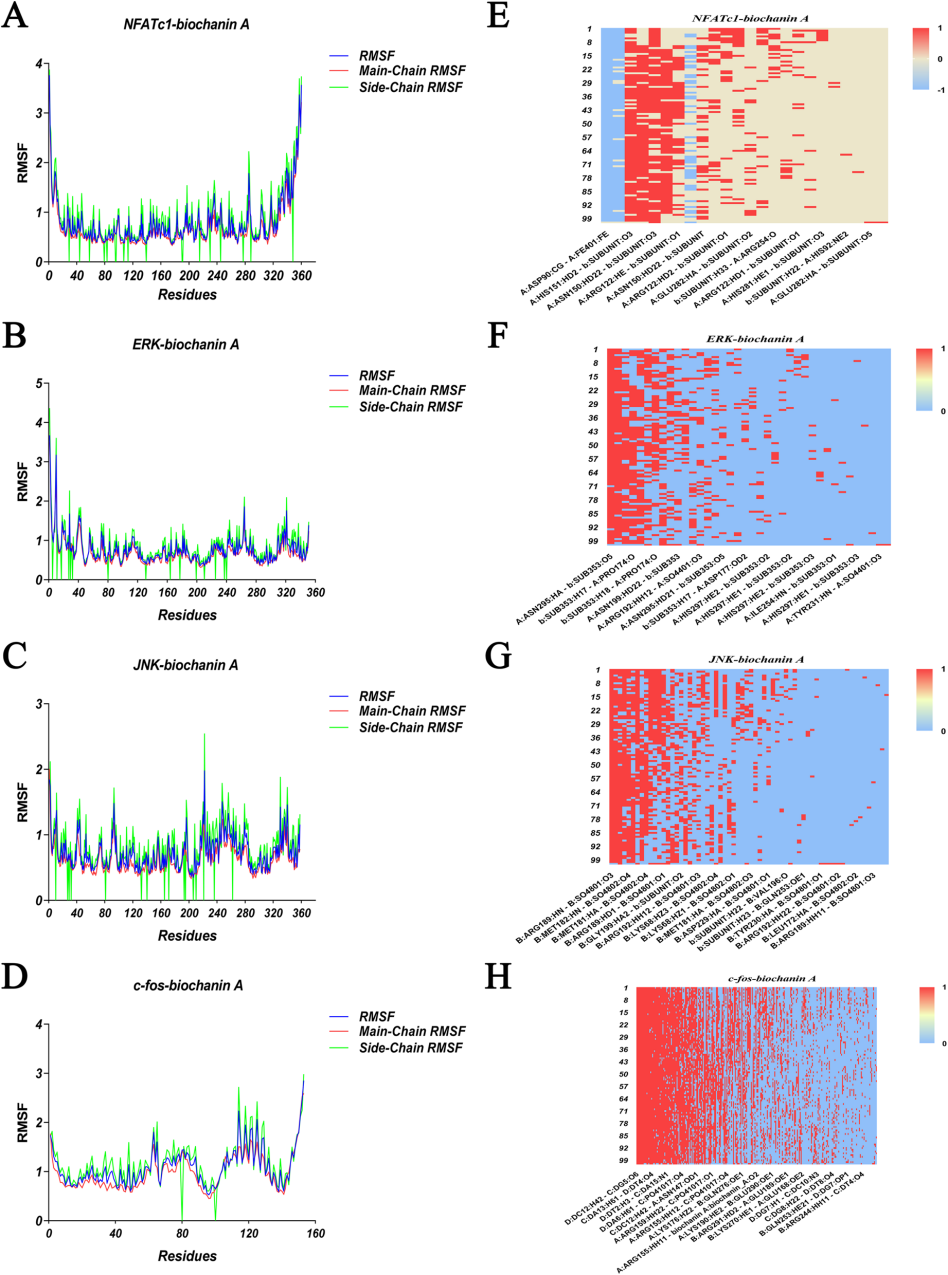
Variation of RMSF values and the Comparison of hydrogen bond interaction heat map of docked complex of (**A**) NFATc1-BCA; (**B**) ERK-BCA; (**C**) JNK-BCA; (**D**) c-FOS-BCA

**Fig. 9:**
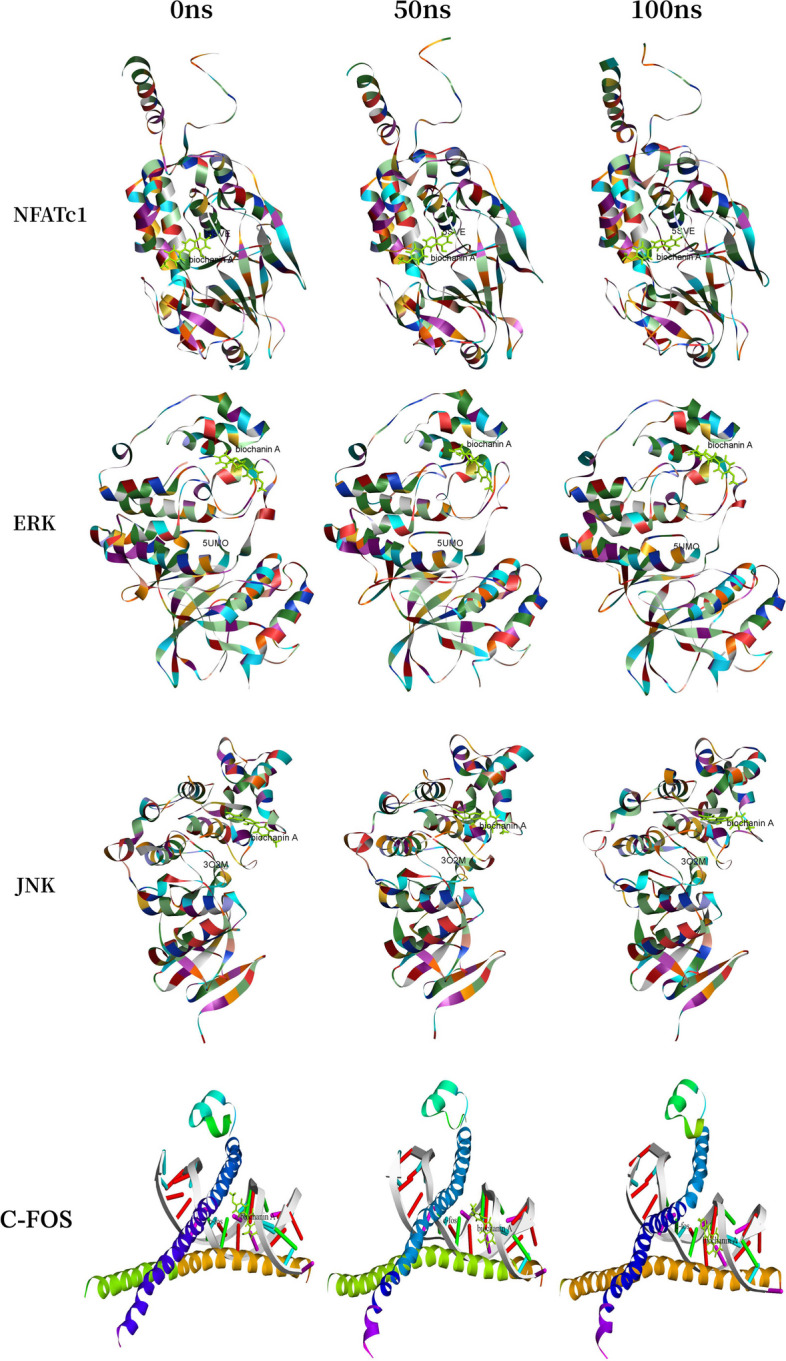
The simulation results of NFATc1-BCA, ERK-BCA, JNK-BCA and c-FOS-BCA receptor-ligand complexes

To investigate the structural stability of the protein-ligand complexes during MD simulations, the RMSD values of the complexes during MD simulations at 100 ns were calculated for the complexes formed during docking. As seen in Fig. 7, the complexes in both systems reached stability after 100 ns of MD simulations. In addition, the RMSD values of NFATc1-BCA complex mainly fluctuated from 1.2014 to 1.72277, with the mean RMSD value of 1.41011 and the total binding energy was calculated to be -119242.326 kcal/mol; the RMSD values of ERK-BCA complex mainly fluctuated from 1.58423 to 2.43306, with the mean RMSD value of 1.99857 and the total binding energy was calculated to be -84871.5556 kcal/mol; and the RMSD values of JNK-BCA complex mainly fluctuated from 1.44074 to 1.86715, with the mean RMSD value of 1.65411 and the total binding energy was calculated to be -90,896.1 kcal/mol; while the RMSD values of c-FOS-BCA complex mainly fluctuated from 1.15361 to 2.53948, with the mean RMSD value of 1.72491 and the total binding energy was calculated to be -112827.207 kcal/mol; The RMSD fluctuations of the three complexes were within the reasonable range, indicating that the structure of the complexes in the system was in a stable state after the simulation. NFATc1-BCA and JNK-BCA complexes were in a stable state during the whole MD simulation process and played a stabilizing role in the formation of the complex. However, the RMSD value of the ERK-BCA complex and c-FOS-BCA complex fluctuated greatly during the 100ns molecular dynamics simulation, which shows its stability was poor. In order to analyze the fluctuation of various amino acids in the complexes during the MD simulation, the RMSF values of all amino acids during the simulation were calculated. The fluctuation trends of the curves of the three complexes were different, i.e., the amino acids of different proteins fluctuated differently during the simulation process, indicating that the formation process of NFATc1, ERK, JNK, and c-FOS in different proteins was different (Fig. 8A). The NFATc1-BCA complex had fluctuations around amino acids Leu11, Ser12, Arg292, Lys293, Ser294, Asn326, Asn327, Val328, the ERK-BCA complex had fluctuations around amino acids Ala5、Ala6、Val12、Arg13、Gly14、Gly30、Glu31、Gly32、Ala323、Glu324、Ala325, and the JNK-BCA complex had fluctuations around amino acids Asp7, Asn8, Asn9, Gly227, Arg228, Asp229, Pro335, Lys336, Ile337, and the c-FOS-BCA complex had fluctuations around amino acids Ala184, Asn185, Leu186, Lys263, His264, His265,Leu266 and the RMSF of amino acid residues in the NFATc1-BCA complex was smaller than that of the JNK-BCA, c-FOS-BCA complex (Fig. 8E). This indicates that the fluctuations of amino acids in the NFATc1-BCA complex are smaller, which plays a role in the stability of the complex. The hydrogen bonding thermograms during molecular docking are shown in Fig. 8D, in the NFATc1-BCA complex, we identified a hydrogen bond interaction formation of 32.38%. For the ERK-BCA complex, the hydrogen bond interaction formation was 21.42%. In the JNK-BCA complex, the hydrogen bond interaction formation accounted for 24.64%. Additionally, the FOS-BCA complex showed a hydrogen bond interaction formation of 28.52%. The simulation results of NFATc1-BCA, ERK-BCA, JNK-BCA, and c-FOS-BCA receptor-ligand complexes are shown in Fig. 9.

### BCA down-regulates RANKL-induced activation of MAPK signaling pathway

**Fig. 10:**
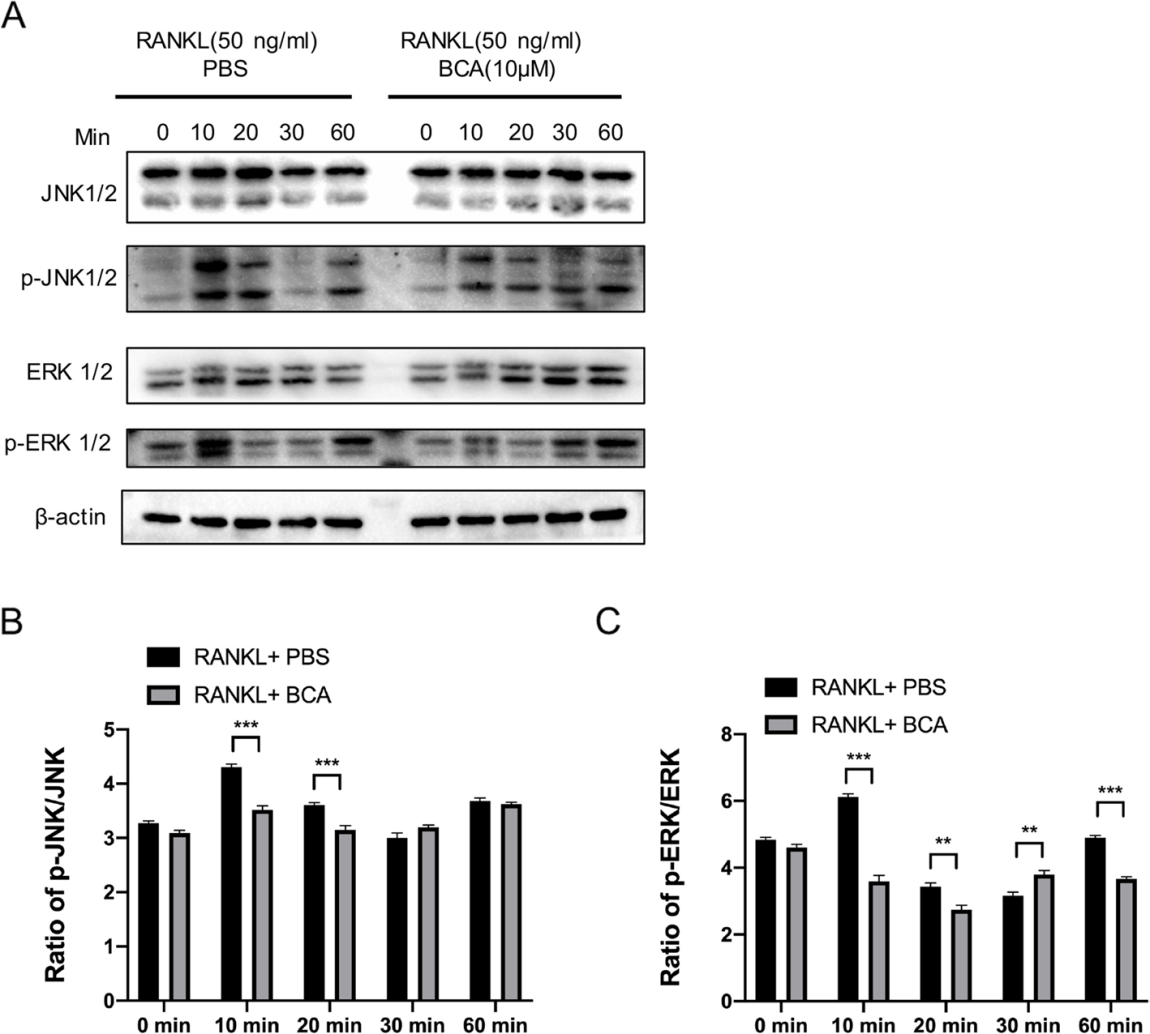
BCA inhibits the RANKL-induced MAPK signaling pathway. **A **Representative western blotting images of the effects of BCA on JNK1/2 and ERK1/2 phosphorylation. BCA was used to pretreat BMMs for 1 h followed by 0, 10, 20, 30 and 60 min of RANKL (50 ng/mL) stimulation. **B**-**C **Quantitative analysis of the fold change in p-JNK and p-ERK expression after BCA (10µM) treatment. Data were presented as the means ± SD; *P < 0.05, and ***P < 0.001 relative to RANKL-induced controls

According to the above results of molecular docking, we speculated that BCA may prevent osteoclast formation in T2DOP through ERK and JNK/MAPK signaling pathway. MAPK signaling pathway is one of the most critical pathways when BMMs transform into OCs. In order to verify whether BCA affects MAPK pathway during osteoclast formation, the expression levels of p-JNK1/2 and p-ERK1/2 proteins were determined by WB at 0, 10, 20, 30, and 60 min following 50 ng/mL RANKL induction in presence and absence of BCA (10µM). At 10 min, BCA exhibited the strongest inhibitory effect on p-ERK1/2 and p-JNK1/2 (Fig. 10A). So, we indicated MAPK signaling pathway is attenuated by BCA via comparing the phosphorylation of ERK and JNK in various time points.

## Discussion

Patients with type 2 diabetes mellitus who are susceptible to osteoporosis mainly show abnormal bone metabolism, increased bone fragility and high risk of bone fractures [26]. Many researches demonstrated that multiple aspects associated with the pathogenesis of T2DOP includes oxidative stress, hyperglycemia and changes in cytokines and hormones [27–30]. However, there are still some shortcomings in the existing research. The therapeutic options of T2DOP are still largely limited and we should search for novel alternative drugs may pave the way to improve the treatment of T2DOP. Various studies indicated that BCA has potential effect in reducing hyperglycemia in types 2 diabetes mellitus [10, 31, 32], but no prior studies have confirmed that BCA can reduce the occurrence of OP induced by T2DM. In our study, we showed for the first time that BCA protects against type 2 diabetic osteoporosis in *db/db* mice that is a model of T2DOP [33] and we found that ROS/MAPK signaling pathway were mainly involved in the treatment of BCA against T2DOP in vitro experiment based on integrating molecular docking.

According to our previous studies, we used *db/db* mice as a model of T2DOP in this experiment [33, 34]. Firstly, we uncovered that bone histology parameters were increased and the intertrabecular space and thickness were improved in the BCA treatment group. This indicates that BCA can inhibit bone loss and reduce the deterioration of trabecular bone microstructure in *db/db* mice as confirmed by micro-CT and H&E staining. Alternatively, the TRAcP staining results showed that BCA could inhibit the number of osteoclasts. Based on these in vivo results, we think that the protective effect of BCA may be through the inhibition of osteoclasts on T2DOP mice.

To validate these in vivo results, further experimental research is required. Molecular docking of osteoclast associated protein receptors with BCA, we found that BCA likely targets NFATc1, ERK and JNK to prevent osteoporosis. In order to better simulate on-body environments and get closer to accurate in vivo performance, BMMs were prepared from the bone marrow of femurs and tibias of *db/db* mice. We found that TRAcP + multinucleated cells formation was significantly promoted by in *db/db* mice. This could be associated with the pathogenesis of diabetic osteoporosis [35]. We firstly demonstrated that BCA could significantly inhibit osteoclast function in vitro without any appreciable cytotoxicity. As is well known, ROS are natural by-products of the normal metabolism of oxygen and are essential in regulating osteoclast differentiation and activity. Besides, we also found that BCA can significantly reduce the production of ROS linked to RANKL-dependent osteoclastogenesis [36, 37].A recent study indicated that ROS has been proven to inhibit MARK phosphatases and consequently enhance MARK expression [38]. The MAPK family, including the ERK, JNK, and P38 three major parallel pathways [39, 40]. And the JNK and ERK are all phosphorylated in response to MAPK signaling pathway stimulation. In our study, our results were further confirmed that the protein expression of phosphorylation of JNK and ERK was suppressed by BCA.

MAPK is intimately related to RANKL-induced osteoclastogenesis by regulating the downstream nuclear factors c-Fos and NFATc1 [41–43]. ERK1/2 induces and activates the downstream target c-Fos, a transcription factor for AP-1, which is essential for osteoclastogenesis [44]. Previous studies have demonstrated that c-Fos is a major component of the transcription factor AP-1, and is an indispensable factor associated with early induction of NFATc1 expression in regulating osteoclast differentiation [45]. Our results showed that NFATc1 and c-Fos activity was remarkably blocked by BCA in RANKL-induced BMMs. Besides, we also found that BCA can downregulate the expression of osteoclastogenesis-related genes and proteins, such as Acp5, V-ATPase-d2, Ctsk, and Mmp9 which are involved in OCs differentiation and function by cooperating with C-Fos and NFATc1 [46, 47]. To sum up, these results confirmed that BCA might be a promising new avenue in treating T2DOP via ROS/MAPK signaling pathway.

## Conclusion

**Fig. 11:**
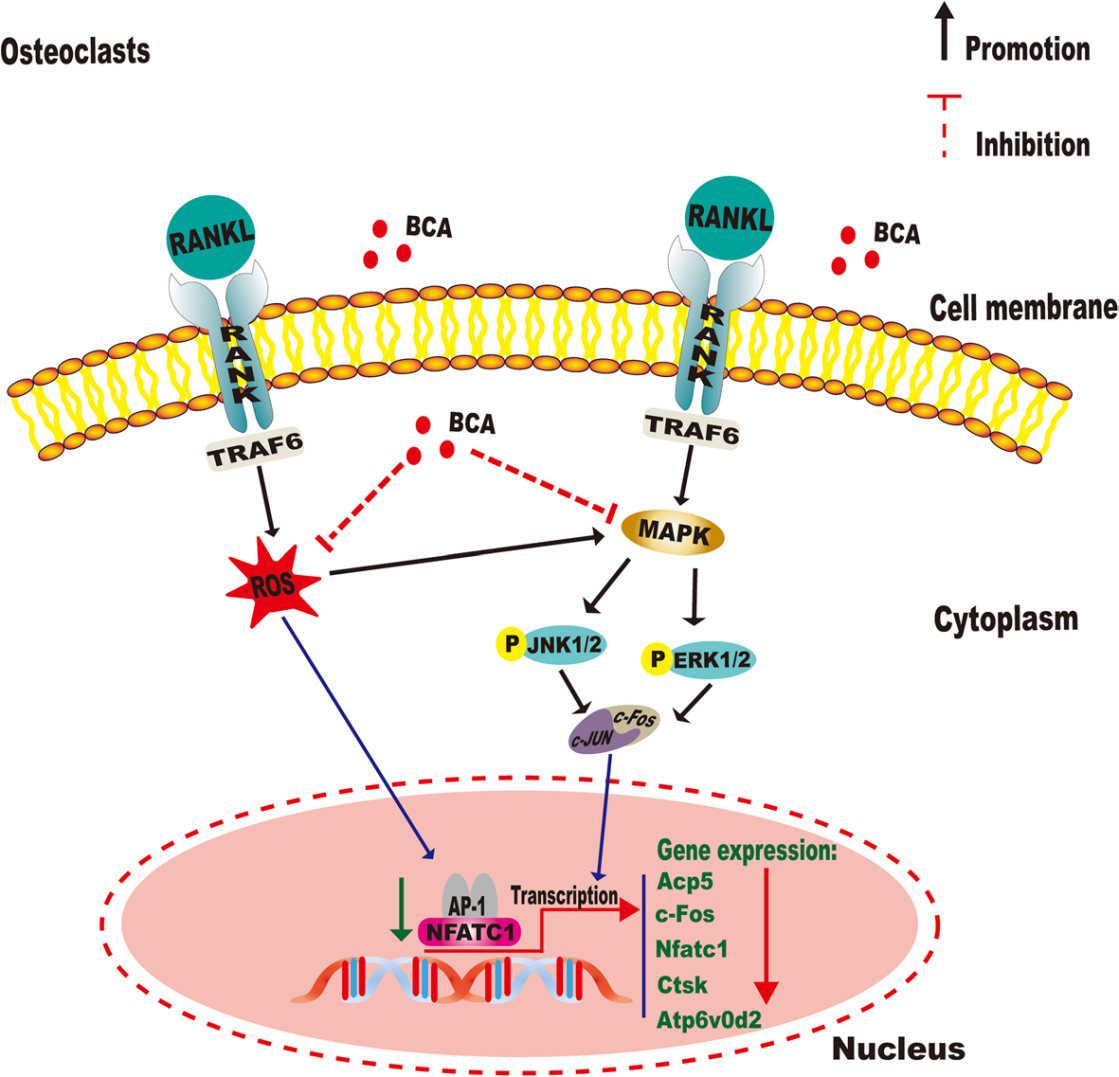
Graphical abstract for illustrating the role of BCA in repressing osteoclastogenesis in type 2 diabetic osteoporosis via regulating ROS/MAPK signaling pathway

We firstly demonstrate that BCA has a significant anti-osteoporosis effect caused by type 2 diabetes and we hope to prove a new treatment strategy for T2DOP. We verified the effect of BCA on osteoclast function by regulating ROS/MAPK signaling pathway in further mechanistic studies (Fig. 11). Although this study revealed the therapeutic effect of BCA on T2DOP mice and also found that BCA may be involved in the regulatory mechanism, there are still some limitations in this study which need to be further studied. Firstly, osteoporosis caused by *db/db* mice is mainly related to the changes in the endogenous hormone leptin level, which may not be applicable to patients with T2DOP caused by other pathological factors. Secondly, we cannot exclude whether the effect of BCA on glucose metabolism also has an anti-osteoporosis effect. Thus, a subsequent study will be required to elucidate this mechanism further.

### Supplementary information

**Additional file 1: Supplementary Table 1. **Physicochemical properties of BCA. **Supplementary Table 2. **Lipophilicity of BCA. **Supplementary Table 3. **Water solubility of BCA. **Supplementary Table 4. **Pharmacokinetics of BCA. **Supplementary Table 5. **Druglikeness of BCA. **Supplementary Table 6. **Medicinal chemistry of BCA. Original gels.

## Data Availability

The PubChem CID of BCA is 5280373 (https://pubchem.ncbi.nlm.nih.gov/compound/5280373). The data supporting this study is open access and can be found in the corresponding databases described in this paper.

## Abbreviations

BP: Biological process
BV/TV: Bone volume/tissue volume
Ct.Ar: Cortical bone area
Ct.Th: Cortical bone thickness
DisGeNET: Disease-gene association database
EDTA: Ethylenediaminetetraacetic acid
ETCM: The Encyclopedia of Traditional Chinese Medicine
GO: Gene Ontology
H&E: Hematoxylin and eosin staining
KEGG: Kyoto Encyclopedia of Genes and Genomes
MF: Molecular function
N.Oc/BS: Osteoclast number/bone surface
Ob.N/BS: Number of osteoclasts
Ob.S/BS: Osteoblast surface ratio
Oc.S/BS: Osteoclast surface/bone surface
OMIM: Online Mendelian Inheritance in Man
qRT-PCR: Quantitative real-time PCR
SHJTT: Sanhuang Jiangtang tablet
SMI: Structure model index
T2DOP: Type 2 diabetic osteoporosis
T2DM: Type 2 diabetes mellitus
Tb.Th: Trabecular thickness
Tb.Sp: Trabecular separation
Tb.N: Trabecular number
TCM: Traditional Chinese Medicine
TRAcP: Tartrate resistant acid phosphatase
VOI: The volume of interest
