# Supplementary material for: Biochanin A abrogates osteoclastogenesis in type 2 diabetic osteoporosis via regulating ROS/MAPK signaling pathway based on integrating molecular docking and experimental validation
Source: BMC Complement Med Ther. 2024 Jan 8;24:24. doi: 10.1186/s12906-023-04332-x (PMC10773052; doi:10.1186/s12906-023-04332-x)

**Supplementary Table 1. physicochemical properties of BCA**

| **Physicochemical Properties** | | | | | | | | | | |
| --- | --- | --- | --- | --- | --- | --- | --- | --- | --- | --- |
| Canonical SMILES | Formula | MW | #Heavy atoms | #Aromatic heavy atoms | Fraction Csp3 | #Rotatable bonds | #H-bond acceptors | #H-bond donors | MR | TPSA |
| COc1ccc(cc1)c1coc2c(c1=O)c(O)cc(c2)O | C16H12O5 | 284.26 | 21 | 16 | 0.06 | 2 | 5 | 2 | 78.46 | 79.9 |

**Supplementary Table 2. lipophilicity of BCA**

| **Lipophilicity** | | | | | |
| --- | --- | --- | --- | --- | --- |
| iLOGP | XLOGP3 | WLOGP | MLOGP | Silicos-IT Log P | Consensus Log P |
| 2.55 | 2.99 | 2.88 | 0.77 | 3.03 | 2.44 |

**Supplementary Table 3. water solubility of BCA**

| **Water Solubility** | | | | | | | |
| --- | --- | --- | --- | --- | --- | --- | --- |
| ESOL Log S | ESOL Solubility (mg/ml) | ESOL Solubility (mol/l) | ESOL Class | Ali Log S | Ali Solubility (mg/ml) | Ali Solubility (mol/l) | Ali Class |
| -3.92 | 3.43E-02 | 1.21E-04 | Soluble | -4.33 | 1.32E-02 | 4.66E-05 | Moderately soluble |

**Supplementary Table 4. pharmacokinetics of BCA**

| **Pharmacokinetics** | | | | | | | | | | | | |
| --- | --- | --- | --- | --- | --- | --- | --- | --- | --- | --- | --- | --- |
| Silicos-IT LogSw | Silicos-IT Solubility (mg/ml) | Silicos-IT Solubility (mol/l) | Silicos-IT class | GI absorption | BBB permeant | Pgp substrate | CYP1A2 inhibitor | CYP2C19 inhibitor | CYP2C9 inhibitor | CYP2D6 inhibitor | CYP3A4 inhibitor | log Kp (cm/s) |
| -5.1 | 2.25E-03 | 7.91E-06 | Moderately soluble | High | No | No | Yes | No | No | Yes | Yes | -5.91 |

**Supplementary Table 5. druglikeness of BCA**

| **Druglikeness** | | | | | |
| --- | --- | --- | --- | --- | --- |
| Lipinski #violations | Ghose #violations | Veber #violations | Egan #violations | Muegge #violations | Bioavailability Score |
| 0 | 0 | 0 | 0 | 0 | 0.55 |

**Supplementary Table 6. medicinal chemistry of BCA**

| **Medicinal Chemistry** | | | |
| --- | --- | --- | --- |
| PAINS #alerts | Brenk #alerts | Leadlikeness #violations | Synthetic Accessibility |
| 0 | 0 | 0 | 2.89 |

**Original gels**

c-Fos


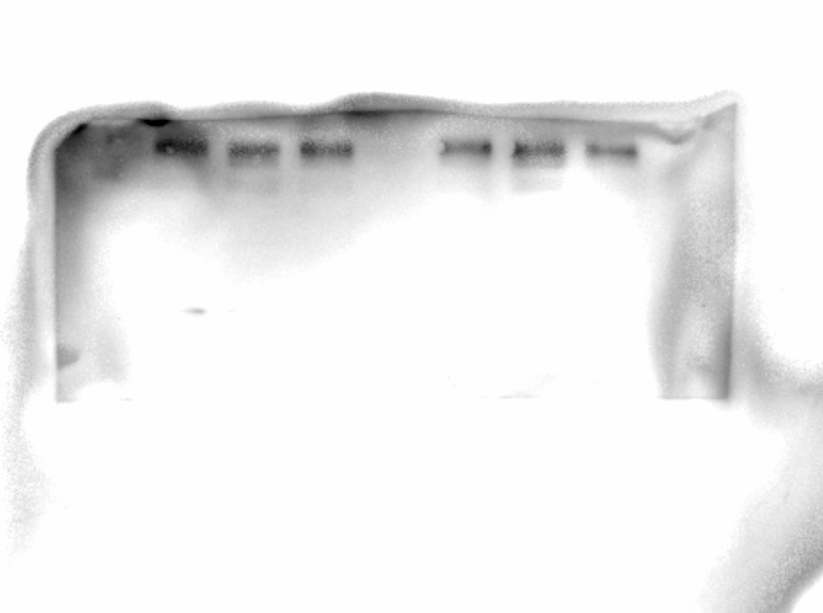

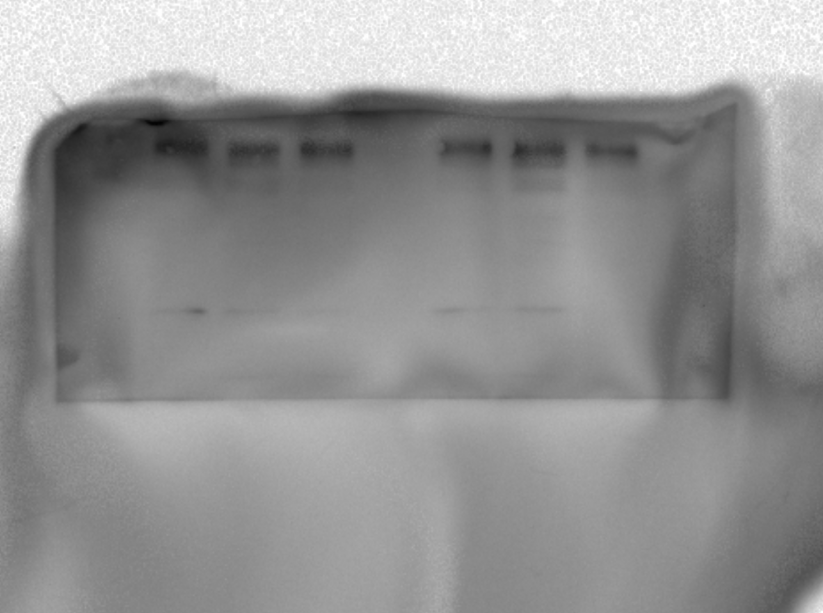


CTSK


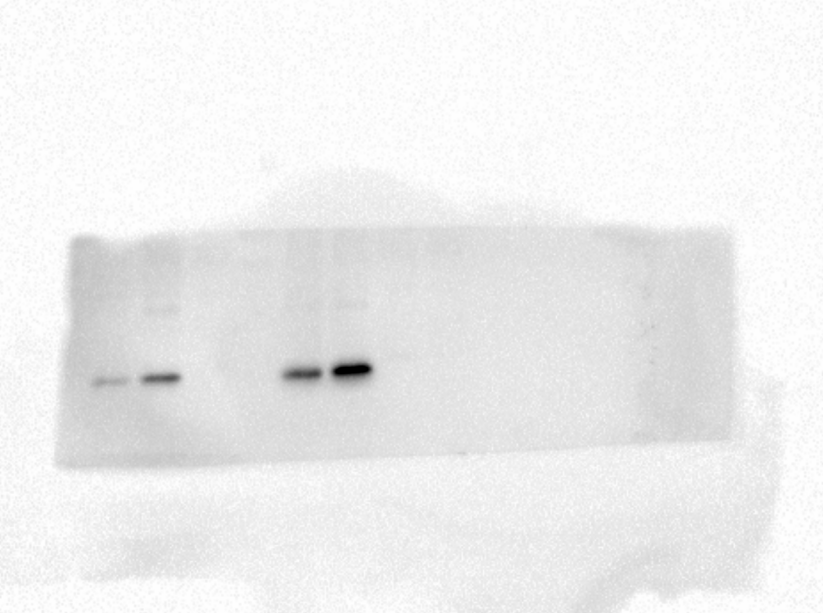

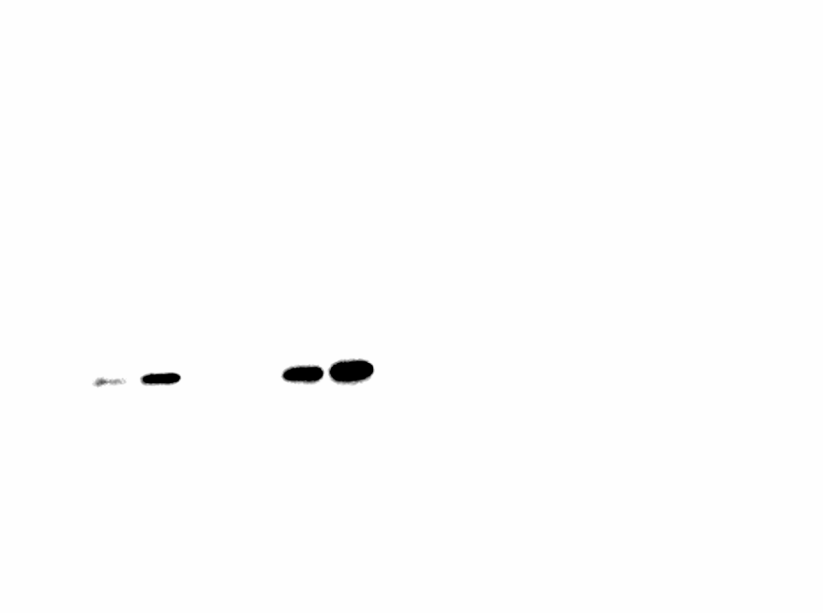


NFATc1


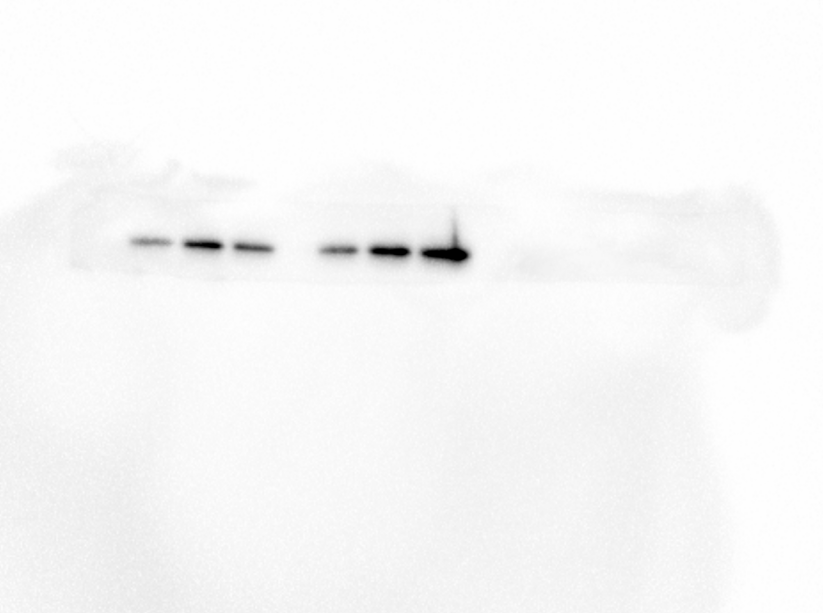


VATPase-d2


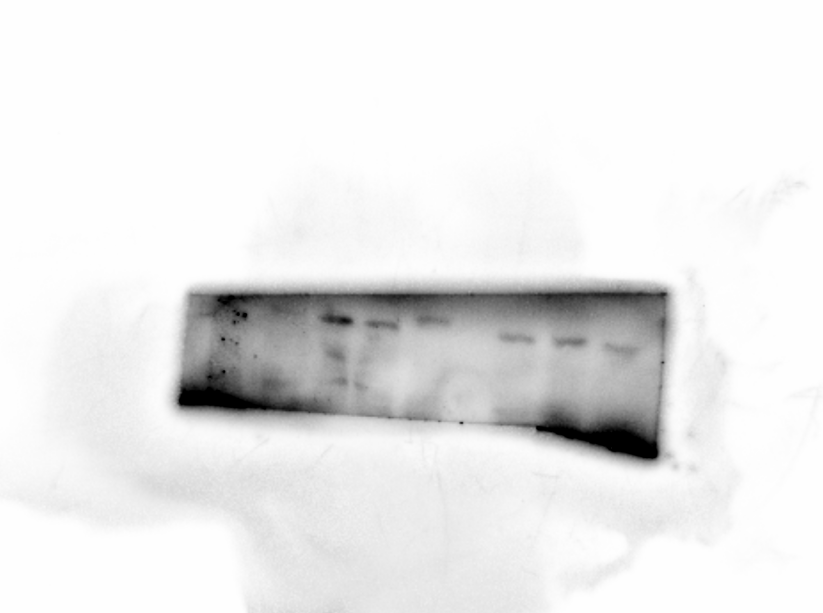

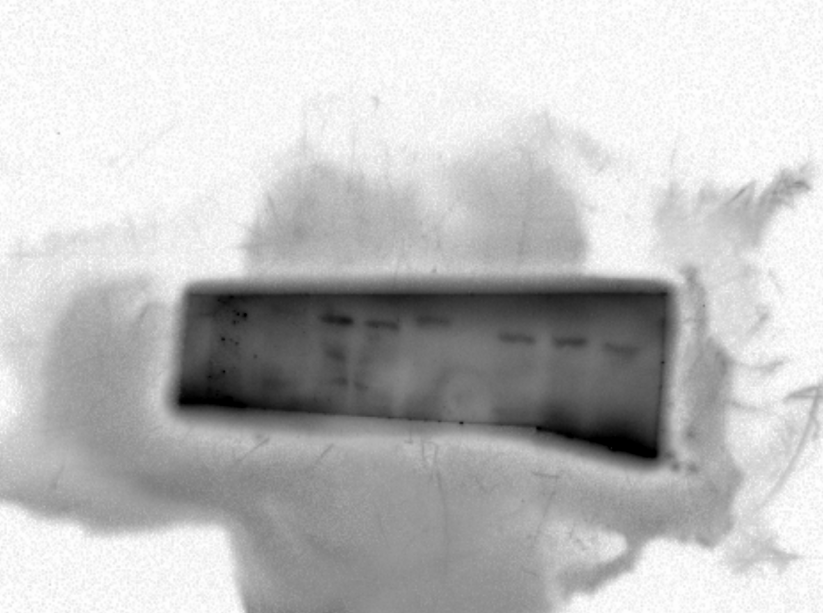


ERK


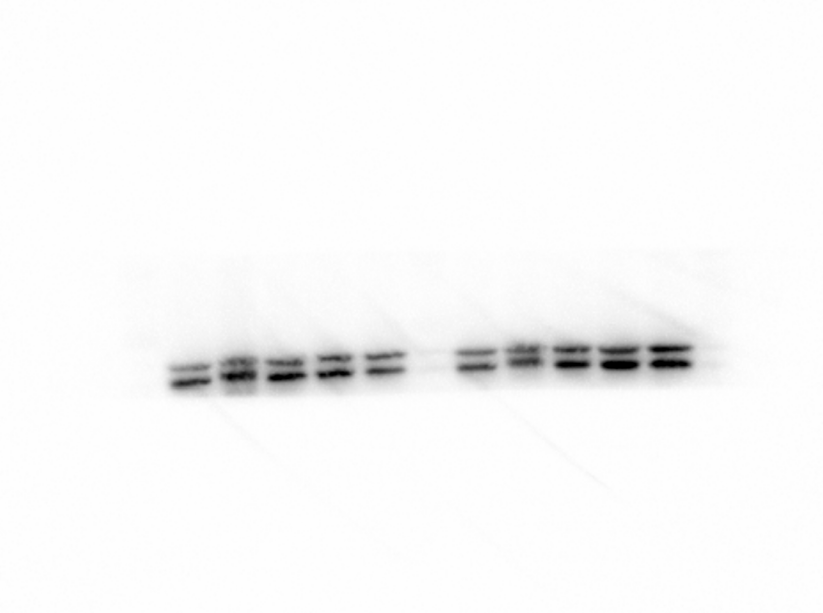


p-ERK


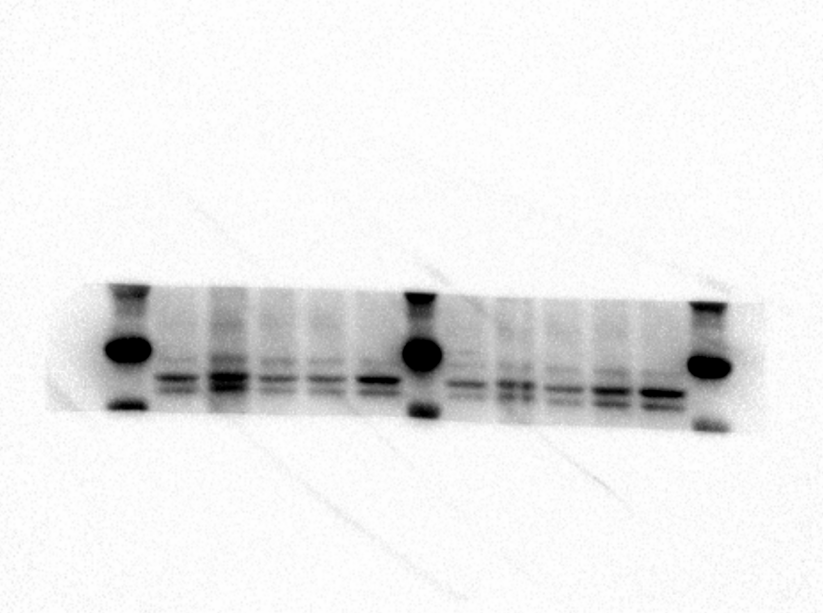


JNK


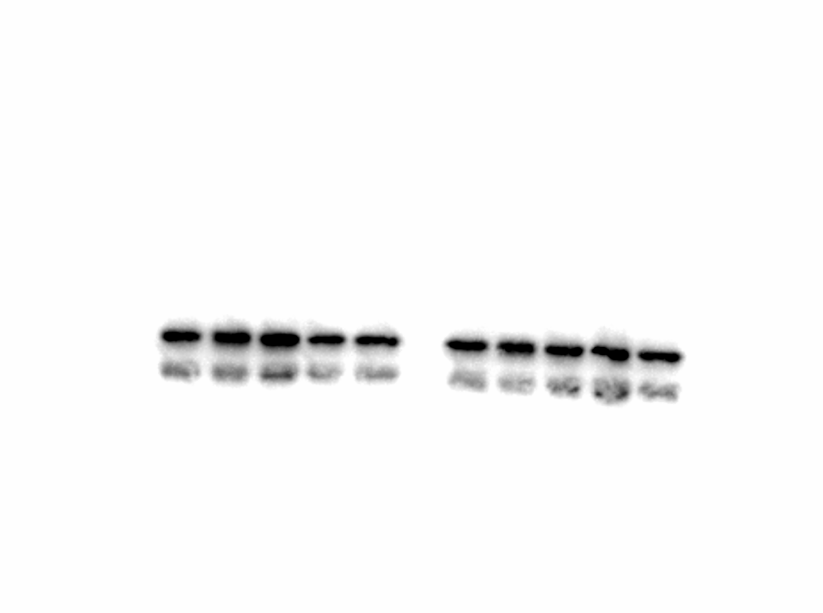


p-JNK


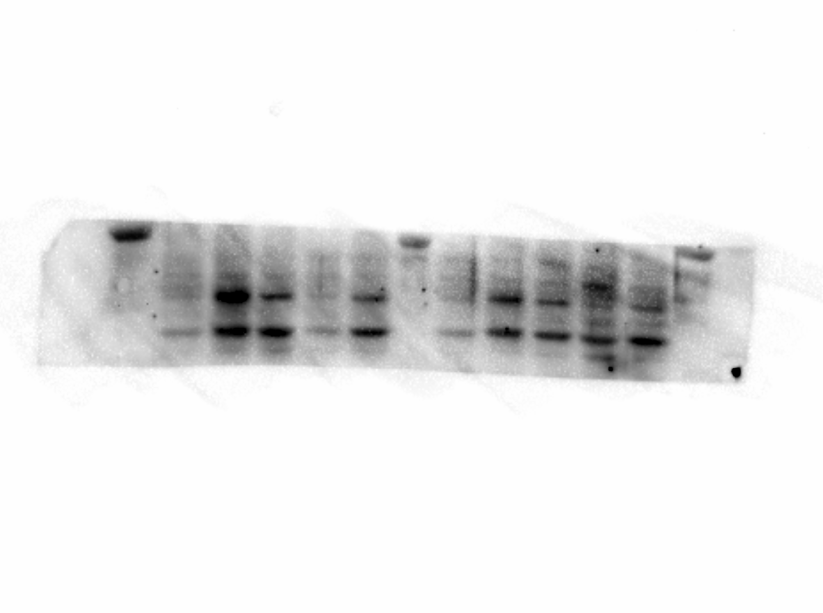


β-Actin


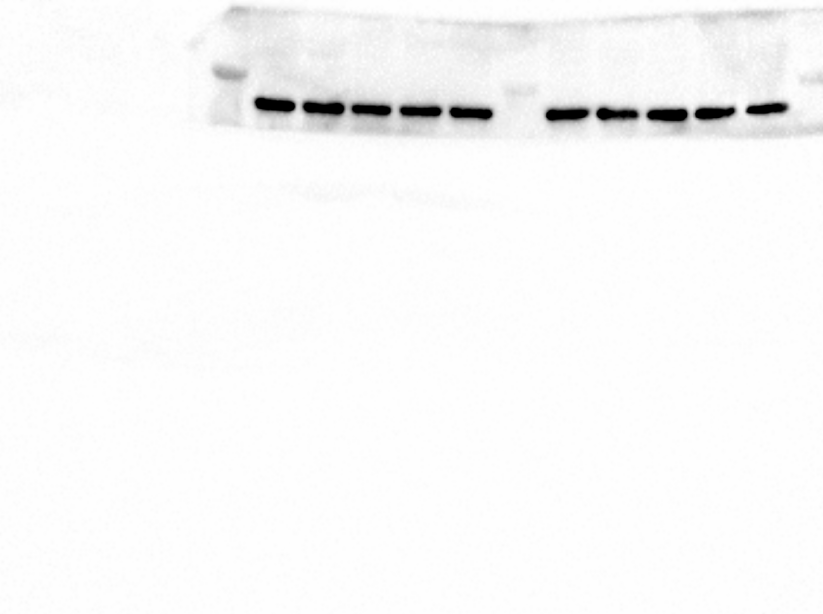

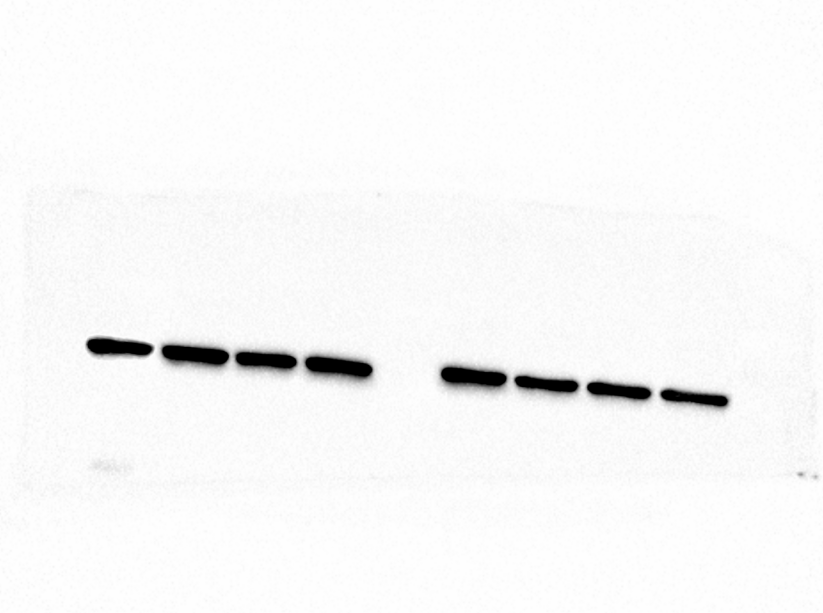

Supplement: Supplementary file 1 — Additional file 1: Supplementary Table 1. Physicochemical properties of BCA. Supplementary Table 2. Lipophilicity of BCA. Supplementary Table 3. Water solubility of BCA. Supplementary Table 4. Pharmacokinetics of BCA. Supplementary Table 5. Druglikeness of BCA. Supplementary Table 6. Medicinal chemistry of BCA. Original gels. [file 12906_2023_4332_MOESM1_ESM.docx]
